# Microvascular retinopathy and angiographically-demonstrated coronary artery disease: A cross-sectional, observational study

**DOI:** 10.1371/journal.pone.0192350

**Published:** 2018-05-08

**Authors:** Lisa Cheng, Peter Barlis, Joel Gibson, Deb Colville, Anastasia Hutchinson, Geoff Gleeson, Ecosse Lamoureux, William VanGaal, Judy Savige

**Affiliations:** 1 The University of Melbourne, Department of Medicine, Northern Health, Melbourne, Australia; 2 Department of Cardiology, Northern Health, Melbourne, Australia; 3 Singapore Eye Research Institute, National University of Singapore, Singapore, Singapore; 4 The University of Melbourne Department of Medicine, Melbourne Health, Melbourne, Australia; Weill Cornell Medicine-Qatar, QATAR

## Abstract

Epidemiological studies suggest retinal microvascular abnormalities predict cardiac events. This study examined microvascular features associated with coronary artery abnormalities. This was a single-centre, cross-sectional, observational study of 144 consecutive subjects undergoing coronary angiography for clinical indications. Their angiograms were deidentified and graded for disease (Leaman score, LAD stenosis ≥ 70%, number of vessels stenosed ≥ 70%), and Thrombolysis in Myocardial Infarction (TIMI) blush score. Subjects also underwent retinal photography (KOWA non-mydriatic camera, Japan), and their deidentified retinal images were graded for hypertensive microvascular retinopathy (Wong and Mitchell classification), vessel calibre using a computer-assisted method (IVAN, U Wisconsin), and diabetic retinopathy (modified Airlie House scheme) independently by a trained grader and an ophthalmologist. Retinal abnormalities were compared between subjects with high and low angiography scores using one way ANOVA, Chi squared and logistic regression analysis (StataCorp, Texas). Subjects had a mean age of 61 years (range 32–88), and included 101 males (70%). Seventeen (12%) had Leaman scores > 10.5, 46 (32%) had LAD stenosis, 13 (9%) had ≥ 3 arteries stenosed, and 20 (14%) had TIMI blush scores < 1. Twenty-six subjects (18%) had a retinal hemorrhage, and 115 (74%) a mild or moderate hypertensive retinopathy. Fifty-five (38%) had diabetes, and 24 (17%) a background (n = 20) or proliferative (n = 4) diabetic retinopathy. A retinal hemorrhage (p = 0.046), moderate microvascular retinopathy (p = 0.08) and proliferative diabetic retinopathy (p = 0.04) were all associated with a higher Leaman score. Venular calibre was increased with triple vessel disease (205.7 ± 21.6 μm, and 193.7 ± 22.3 μm in normals, p = 0.03). Diabetic retinopathy correlated with an increased TIMI blush score (p = 0.01). Retinal microvascular imaging warrants further evaluation in identifying the presence, extent and nature of coronary artery disease.

## Introduction

Coronary artery disease is a major cause of death worldwide [1], and, in general, coronary angiography indicates the extent and immediacy of the risk. However angiography also has limitations in that it does not detect the 20% of individuals with normal angiograms [2, 3]. who have subendocardial ischemia [4] due to microvascular disease [5–8].

The retinal microvasculature reflects pathology in the systemic small vessels including the coronary microcirculation. In general, risk factors for vascular disease are common to both the coronary arteries and the coronary microcirculation, which means that the retinal microvasculature may also indicate the likelihood of pathology in the coronary arteries.

Previous studies have confirmed that retinopathy features correlate with cardiac risk. Microvascular retinopathy is associated with increased coronary heart disease [9–12], and hemorrhages, exudates and cottonwool spots with an increased cardiac mortality [10, 13]. Changes in small vessel calibre predict cardiac events in epidemiological studies [14]. Retinal arteriole narrowing correlates with coronary disease in women, and stroke in men and women [15]. Retinal venular dilatation is also associated with increased cardiac risk [15]. Features of diabetic retinopathy are also associated with increased cardiac mortality [16].

Retinal imaging and microvascular grading is a potential tool to assess both coronary artery and microvascular disease [17]. Retinal imaging and grading is fast, accurate, non-invasive, inexpensive, widely-available and acceptable to patients [18].

This study assessed hypertensive and diabetic microvascular retinopathy features including vessel calibre in subjects with coronary artery and small vessel disease demonstrated on coronary angiography.

## Methods

### Study design

**T**his was a single centre, cross-sectional observational study of subjects recruited consecutively from a Melbourne metropolitan teaching hospital (Northern Health) over a 6 month period.

Recruitment, data capture, coronary angiography and retinal photography were coordinated in a single episode. In general, subjects were studied two hours after angiography, and assisted to complete a structured questionnaire of vascular risk factors. Deidentified angiograms were assessed for Leaman score, diseased vessel score, LAD stenosis ≥70%, and TIMI blush score by a cardiologist and a trained observer.

Subjects also underwent retinal photography, and deidentified images were examined for microvascular and diabetic retinopathy, and for vessel calibre.

The primary outcome was an association of hypertensive or diabetic microvascular retinopathy or vessel calibre with a higher Leaman (incorporating ≥ 70% LAD stenosis, diseased vessel score) or TIMI blush score. Secondary outcomes were the association of individual retinopathy features with a higher Leaman score or TIMI blush score. There were no changes to the study design after its commencement and no interim analyses.

This project was approved by the Human Research Ethics Committee of Northern Health according to the Declaration of Helsinki, and all participants provided, written, informed consent.

### Subjects

Consecutive subjects undergoing non-emergency coronary angiography on the study days over a 6month period were invited to participate. Subjects with ungradeable retinal images were excluded.

### Measurements

#### Clinical features

Study subjects were assisted to complete a structured questionnaire for demographics (age, gender, race), vascular risk factors (hypertension, diabetes, smoking history, dyslipidemia), and laboratory test results (haemoglobin, eGFR) were obtained from the electronic medical records. Hypertension diagnosis was based on a previous physician-made diagnosis (BP≥ 140/90 mm Hg). Past and current smokers were considered to have a smoking history, and pack years noted. Body mass index was calculated from weight in Kg/ (height in m)^2^.

#### Coronary angiography

Subjects underwent left heart catheterisation and coronary angiography using a size 5 or 6 French catheter by a cardiologist according to a standard protocol. Coronary angiograms were stored using Xcelera Cath software (Philips, Nevada) and data extracted in standard DICOM viewing format. Angiograms were deidentified, and a reported by one cardiologist who had undertaken the procedure. The Leaman score for each angiogram was then graded by a cardiologist (PB). The diseased vessel score, and LAD stenosis ≥ 70% were noted from the original reports by the researcher (LC) and confirmed by a physician (JS). The Leaman score assessed the severity of coronary artery disease [19]. LAD stenosis ≥ 70% was noted. The diseased vessel score [20] indicated the number of major coronary arteries with a severe stenosis (≥ 70%) (scores 0, 1, 2 or 3). Lesions in 7 coronary arteries were recorded: left main coronary, left anterior descending, left circumflex, right coronary, left diagonal, obtuse marginal, or a graft artery. Scores were assigned to each lesion based on location and severity, with greater weight given to more proximal and more severe stenoses. The sum of the scores was calculated to obtain the total coronary score, with higher scores indicating more severe disease [19, 21].

Each deidentified angiogram was also assessed for a TIMI blush score or the intensity of the radioopacity of myocardial tissue and the rapidity with which it cleared by one cardiologist (PB)[6, 22]. Each lesion was assigned a score of 0 to 3, with lower scores indicating worse perfusion.

#### Retinal imaging and grading

All subjects underwent digital retinal imaging of both optic fundi, using a non-mydriatic retinal camera (KOWA 7, KOWA Japan). Standard 30^o^ images centred on the macula or optic disc were taken. Deidentified images were examined for hemorrhage (on red-free images) and graded for microvascular retinopathy (Wong and Mitchell classification, [9]) and diabetic retinopathy (modified Airlie House grading scheme [23]) independently by a trained grader (LC) and by an ophthalmologist (DC). Any differences in grade were discussed until a consensus was reached, and where there was a no consensus, the opinion of the ophthalmologist was adopted. An overall grade was assigned to each subject based on the eye with the more severe retinopathy. The following definitions were used in grading. ‘Generalised arteriolar narrowing’ was defined as an arteriovenous ratio less than two-thirds; ‘focal arteriolar narrowing’ when an arteriole ≥50 μm (one third the diameter of a major vein at the optic disc margin) had a constricted area of two-thirds or less of the width of proximal and distal vessel segments; and ‘arteriovenous nicking’ was defined as an arteriovenous crossing where the venous blood column was tapered on both sides of an arteriole.

Retinal vessel calibres were measured by a trained grader at the Centre for Eye Research Australia using a standardised protocol [24, 25]. Briefly, all vessels passing through a region 0.5 to 1 disc diameter from the optic disc were measured using a computer imaging programme (University of Wisconsin, WI), and summary measures based on the 6 largest vessels combined into the Central Retinal Artery Equivalent (CRAE) and Central Retinal Vein Equivalent (CRVE) using Knudtson’s modification of the Parr-Hubbard formula [25, 26]. This method is highly reproducible with intraclass correlation coefficients of 0.99 for arterioles and 0.94 for venules.

#### Arteriole and venular calibre pre-and post-angiography

Because of concerns that retinal small vessel calibre might change with medications administered during angiography, a further cohort of subjects were studied with retinal imaging performed both before and after angiography.

### Statistical analysis

The relationships between retinal hemorrhage, microvascular and diabetic retinopathy and: Leaman score, (incorporating LAD stenosis, diseased vessel score) and TIMI blush score were assessed using one way analysis of variance (ANOVA). Multivariate analysis was performed using logistic regression for dichotomous outcomes, multiple regression for continuous outcomes and ordered logistic regression for grouped outcomes. Odds ratios and p values were obtained for each variable.

Retinal arteriole and venular calibre measurements were categorised by quartiles, and differences between subjects with calibre in the lowest and highest quartiles compared using the Chi-squared test or ANOVA.

All statistical analyses were performed using STATA version 10 software (StatCorp, College Station, Texas). A p value of < 0.5 was significant and a value < 0.10 was considered a trend.

## Results

### Baseline characteristics of study subjects

One hundred and forty-five subjects were recruited. One was excluded because his retinal images were ungradeable for retinopathy, resulting in a total of 144.

Subjects had a median age of 61 years (range 32–88), and included 101 males (80%), 101 individuals with hypertension (80%), 55 (38%) with diabetes, 130 (90%) with dyslipidemia or undergoing statin treatment, 109 (76%) current or former smokers (mean of 24 ± 30pack years), with an overall mean BMI of 30 ± 5.4 kg/m^2^, mean Hb of 138 ± 16.7 g/l and mean eGFR of 74 ± 17 ml/min/1.73 m^2^ (range 24–90) **(Table 1)**.

**Table 1:** Clinical features, retinal abnormalities and Leaman scores.

Characteristics	Leaman score >10.5		Leaman score >6.5 to 10.5		Leaman score >0 to 6.5		Leaman score 0		*P* Value
	n = 17		n = 30		n = 54		n = 43		
	Mean (SD)	%	Mean (SD)	%	Mean (SD)	%	Mean (SD)	%	
Age, years	63 (14)		60 (10)		64 (10)		59 (11)		0.07
Male		88		79		72		56	**0.04**
Dyslipidaemia		100		97		94		78	**<0.01**
Hypertension		71		83		77		53	**0.02**
Diabetes		29		59		47		18	**<0.01**
BMI, kg/m^2^	28 (3)		30 (5)		30 (6)		31 (6)		0.40
Smoking		82		86		74		69	0.33
Smoking pack years	32 (40)		27 (35)		24 (31)		19 (21)		0.43
Hb, g/L	135 (22)		137 (16)		137 (16)		142 (16)		0.40
eGFR, mL/minute/1.73m^2^	65 (25)		74 (18)		72 (17)		79 (11)		**0.02**
**Retinal abnormalities**									
Hemorrhage		47		17		13		14	**0.01**
Exudates		12		14		17		16	0.95
Any microvascular retinopathy		88		62		77		71	0.23
Moderate /severe microvascular retinopathy		53		24		23		22	0.08
Any diabetic retinopathy		29		21		19		7	0.12
Moderate/proliferative diabetic retinopathy		0		10		0		2	**0.04**
Arteriolar calibre (μm)	134.2 (12.5)		134.8 (13.0)		131.7 (10.6)		133.6 (15.1)		0.45
Venular calibre (μm)	199.7 (17.8)		197.5 (20.1)		199.3 (18.7)		194.1 (22.2)		0.31

*P* value represents the difference in characteristic by analysis of variance or the chi-square test.

All subjects were currently treated with aspirin, and sometimes clopidigrel. Twenty (14%) had retinal images before angiography and 124 (86%) within the subsequent four hours.

### Retinal abnormalities

Twenty-six subjects (18%) had a retinal hemorrhage, 22 (15%) had exudates, and two (2%) had cholesterol emboli.

#### Microvascular retinopathy

One hundred and five individuals (73%) had a microvascular retinopathy, with mild changes in 67 (73%), and moderate in 38 (27%). Thirty-nine (27%) had no retinopathy and none had severe changes. Their mean arteriole calibre (CRAE) was 133.4 ± 11.8 μm and mean venular calibre (CRVE) was 197.4 ± 18.6 μm.

#### Diabetic retinopathy

Fifty-five individuals had diabetes, 24 (17%) of whom had a diabetic retinopathy, with background changes in 20 (14%) and moderate-proliferative changes in 4 (3%).

### Coronary artery assessment

#### Leaman score

One hundred and one subjects (70%) had an increased Leaman score: 54 (38%) had a score of > 0 to 6.5, 30 (21%) > 6.5 to 10.5 and 17 (12%) > 10.5 **(Table 1)**. Their mean score was 5.0 ± 5.7, and median was 3.0, range 0–30. Forty-three subjects (30%) had a score of 0.

The Leaman score was higher in individuals who were male (0.04), or had hypertension (p = 0.02), diabetes (p = 0.001), dyslipidemia (p<0.01), or an eGFR < 90ml/min/1.73 m^2^ (p = 0.02). There was an association with increasing age (p = 0.07).

Retinal hemorrhage was more common in subjects with a higher Leaman score (p = 0.01). Fifty-three % of individuals with a score > 10.5 and 22% of those with a score of 0 had a moderate microvascular retinopathy (p = 0.08). Twenty-nine % of those with a Leaman score > 10.5 but only 7% with a score of 0 had a diabetic retinopathy (p = 0.12). Four individuals had a moderate diabetic retinopathy, three of whom had a Leaman score of > 6.5 to 10.5 and one with a score of 0 (p = 0.04).

There was no difference in retinal arteriolar calibre between subjects with a high or 0 Leaman score (134.2 ± 12.5 μm and 133.6 ±15.1 μm, p = 0.45). There was also no difference in venular calibre (199.7 ± 17.8 μm and 194.1 ± μm, p = 0.31).

Forty-six subjects (32%) had an LAD stenosis ≥ 70%. Retinal hemorrhage, microvascular and diabetic retinopathy did not correlate with a significant stenosis (p = 0.25, 0.84, p = 0.12 respectively). There were only four subjects with a proliferative diabetic retinopathy, three of whom had severe artery stenoses (p = 0.09). There was no difference in retinal arteriole or venular calibre in individuals with or without a severe LAD stenosis (p = 0.88, p = 0.59 respectively).

When angiograms were assessed for diseased vessel score, 13 subjects (9%) had 3 or more diseased coronary vessels, 26 (18%) had two, 63 (43%) had one and 42 (29%) had none. Retinal hemorrhage was more common in individuals with 3 or more diseased vessels (p = 0.046). Otherwise microvascular retinopathy and diabetic retinopathy were not associated with the number of diseased coronary vessels (p = 0.34, 0 = 0.15 respectively). Arteriolar calibre was not different with an increased number of diseased coronary vessels. However retinal venular calibre was larger in subjects with more diseased arteries (205.7 ± 21.6 μm in those with 3 or more compared with 193.2 ± 21.1 μm in those with none, p = 0.03).

When multivariate analysis using ordered logistic regression was performed to determine if the grade of microvascular retinopathy was associated with Leaman score (> 0 to 6.5, > 6.5 to 10.5, > 10.5), the model demonstrated that microvascular retinopathy grade predicted the Leaman score (OR 1.55, 95% CI 0.97 to 2.47, p = 0.067) after controlling for traditional cardiac risk factors including age, gender, diabetes, dyslipidemia, hypertension and renal impairment **(Table 2)**. A similar model demonstrated that diabetic retinopathy was associated with the Leaman score (OR 1.81, 95% CI 1.05 to 3.10, p = 0.03) after adjusting for the same factors **(Table 2)**.

**Table 2:** Multivariate model for Leaman score prediction including clinical features and retinal abnormalities.

	Microvascular retinopathy			Diabetic retinopathy		
	Odds ratio	95% confidence level	P value	Odds ratio	95% confidence level	P value
**Age (years)**	1.00	0.97 to 1.03	0.98	1.00	0.97 to 1.04	0.94
**Gender**	3.88	1.85 to 8.15	<0.01	4.55	2.12 to 9.79	<0.01
**Hypertension**	1.84	0.88 to 3.82	0.10	1.97	0.95 to 4.11	0.069
**Diabetes**	1.33	0.69 to 2.59	4.00	1.26	0.64 to 2.46	0.51
**Dyslipidemia**	5.77	1.58 to 21.05	0.01	6.13	1.68 to 22.34	0.01
**eGFR (ml/min/1.73 m**^**2**^**)**	0.98	0.95 to 1.00	0.02	0.98	0.96 to 1.00	0.046
Any abnormal microvascular (1^st^) or diabetic (2^nd^ columns) retinopathy	1.55	0.97 to 2.47	0.067	1.81	1.05 to 3.10	0.03

When ROC curves were plotted, and sensitivity and specificity calculated for individual variables, to determine their utility in predicting a Leaman score > 6.5, all variables had area under the curves (AUC) close to 0.5, demonstrating little discriminative ability. When ROC curves were calculated for different multivariate models to determine their utility in predicting a Leaman score > 6.5, the AUC for one model (with traditional risk factors, age, gender, diabetes, dyslipidemia, eGFR and hypertension) was 0.72 demonstrating fair to good discriminative ability. When microvascular retinopathy or diabetic retinopathy were added to the model, there was a 1.0% or 1.3% increase in AUC respectively. Thus including retinopathy in the risk assessment produced no benefit.

#### TIMI blush score

The score was averaged for one to 4 stenoses in the 7 coronary arteries examined. Sixty-three subjects (44%) had a normal TIMI blush score and 81 (56%) had an abnormal score **(Table 3)**. The mean TIMI blush score was 1.7 ± 1.0, with a median of 2.0, range 0–3). An abnormal score was more common in males (p = 0.048), and individuals with dyslipidemia (p<0.046), and there were trends with hypertension (p = 0.07), and diabetes (p = 0.07).

**Table 3:** Clinical features, retinal abnormalities and TIMI blush scores.

Characteristic	Subjects with TIMI Blush score <1 (severe)		Subjects with TIMI Blush score 1 to <2		Subjects with TIMI Blush score 2 to <3		Subjects with TIMI Blush score of 3		P Value
	n = 20		n = 18		n = 43		n = 63		
	Mean (SD)	%	Mean (SD)	%	Mean (SD)	%	Mean (SD)	%	
Age, years	62 (8)		61 (13)		63 (11)		60 (11)		0.61
Male		85		83		74		59	**0.048**
Dyslipidaemia		95		100		95		83	**0.046**
Hypertension		75		77		81		59	**0.07**
Smoking		85		89		74		70	0.28
Smoking pack years	31 (34)		19 (11)		30 (41)		20 (24)		0.26
Hb, g/L	136 (15)		134 (17)		137 (18)		141 (16)		0.27
Diabetes		55		39		47		27	**0.07**
BMI, kg/m^2^	31 (5)		31 (5)		30 (5)		30 (6)		0.70
eGFR, mL/minute/1.73m^2^	73 (21)		69 (20)		71 (19)		77 (13)		0.14
**Retinal abnormalities**									
Hemorrhage		25		22		19		15	0.71
Exudates		10		11		21		15	0.63
Any abnormal microvascular retinopathy		70		78		79		70	0.73
Moderate or severe microvascular retinopathy		15		28		19		24	0.74
Any abnormal diabetic retinopathy		20		17		31		6	**0.01**
Moderate to proliferative diabetic retinopathy		0		6		5		2	0.57
Arteriole calibre (μm)	136.1 (19.4)		133.8 (12.9)		131.1 (13.0)		133.7 (14.1)		0.33
Venular calibre (μm)	200.0 (18.2)		198.3 (17.2)		194.2 (18.5)		198.2 (22.1)		0.51

Retinal hemorrhage and microvascular retinopathy were not increased in subjects with a higher TIMI score **(Table 3)**. However a diabetic retinopathy was present in 20% of individuals with a TIMI blush score of <1 and only 6% of those with a TIMI blush score of 3 (normal) (p = 0.01). The association with a diabetic retinopathy was strong with an OR of 4.92 (CI 1.58 to 15.25, p = 0.003).

When multivariate analysis using ordered logistic regression was performed to determine if the grade of microvascular retinopathy were associated with an abnormal TIMI blush score, there was no association (OR 1.68, CI 0.79 to 3.55, p = 0.18) when other traditional risk factors were taken into account **(Table 4)**. A multivariate model using logistic regression was used to determine whether the grade of diabetic retinopathy was associated with an abnormal TIMI blush score. This demonstrated that diabetic retinopathy grades correlated with an abnormal blush score (OR 9.92, 95%CI 1.03 to 95.86, p = 0.047) when other risk factors were taken into account. This relationship was even stronger when any diabetic retinopathy was considered rather than severity. This demonstrated that a diabetic retinopathy predicted an abnormal blush score even more than other criteria (OR 11.99, 95%CI 1.26 to 113.96, p = 0.03).

**Table 4:** Multivariate model for Leaman score prediction including clinical features and retinal abnormalities.

	Hypertensive retinopathy			Diabetic retinopathy		
	Odds ratio	95% confidence level	P value	Odds ratio	95% confidence level	P value
**Age (years)**	0.95	0.90 to 1.01	0.09	0.94	0.89 to 1.00	0.06
**Gender**	2.76	0.78 to 9.77	0.12	3.44	0.93 to 12.80	0.07
**Hypertension**	2.37	0.71 to 7.83	0.16	3.43	0.98 to 12.01	0.05
**Diabetes**	0.94	0.32 to 2.71	0.91	0.80	0.27 to 2.36	0.68
**Dyslipidemia**	0.63	0.05 to 7.67	0.71	0.52	0.04 to 6.61	0.62
**eGFR (ml/min/1.73 m**^**2**^**)**	0.98	0.94 to 1.01	0.24	0.99	0.95 to 1.02	0.46
Any abnormal microvascular (1^st^) or diabetic (2^nd^ columns) retinopathy	1.68	0.79 to 3.55	0.18	11.99	1.26 to 113.96	0.03

ROC curve analysis demonstrated that both microvascular retinopathy and diabetic retinopathy produced an AUC close to 0.65 demonstrating close to fair discriminative ability to predict an abnormal TIMI blush score **(Table 5)**. Both forms of retinopathy were better predictors than most other traditional risk factors when used alone. When four different models with traditional risk factors, and then with microvascular retinopathy or diabetic retinopathy were considered, the traditional risk factors (age, gender, diabetes, dyslipidemia, eGFR, hypertension) produced an AUC of 0.72. This was not improved when microvascular retinopathy was added (AUC = 0.72), but was improved with the addition of diabetic retinopathy (AUC = 0.76).

**Table 5:** Sensitivity, specificity and likelihood ratios of individual variables for predicting an abnormal TIMI blush score.

	Area under ROC curve	Sensitivity	Specificity	Likelihood ratio +	Likelihood ration -
**Gender**	0.56	79%	33%	1.19	0.63
**Hypertension**	0.56	79%	33%	1.19	0.63
**Diabetes**	0.52	47%	57%	1.09	0.93
**Dyslipidemia**	0.51	96%	5%	1.01	0.78
**Microvascular retinopathy**	0.62	77%	48%	1.46	0.49
**Diabetic retinopathy**	0.60	25%	95%	5.25	0.79

There was no difference in retinal arteriole or venular calibre in individuals with an abnormal or normal TIMI blush score (p = 0.33, p = 0.51 respectively).

#### Retinal arteriole and venular calibre

Retinal arteriole calibre was smaller in older individuals (p = 0.0001), and those with hypertension (p = 0.01) or dyslipidemia (p = 0.047). They were also smaller in individuals with microvascular retinopathy but did not correlate with Leaman score, any component of the Leaman score, nor with an abnormal TIMI blush score (although this was close with a p = 0.12).

Likewise, retinal venular calibre was less in older individuals (0.001), and those with a lower eGFR (p = 0.04). Venular calibre did not correlate with the presence of microvascular or diabetic retinopathy, nor with worse Leaman or TIMI blush score.

#### Arteriole and venular calibre pre-and post-angiography

**Fig 1:**
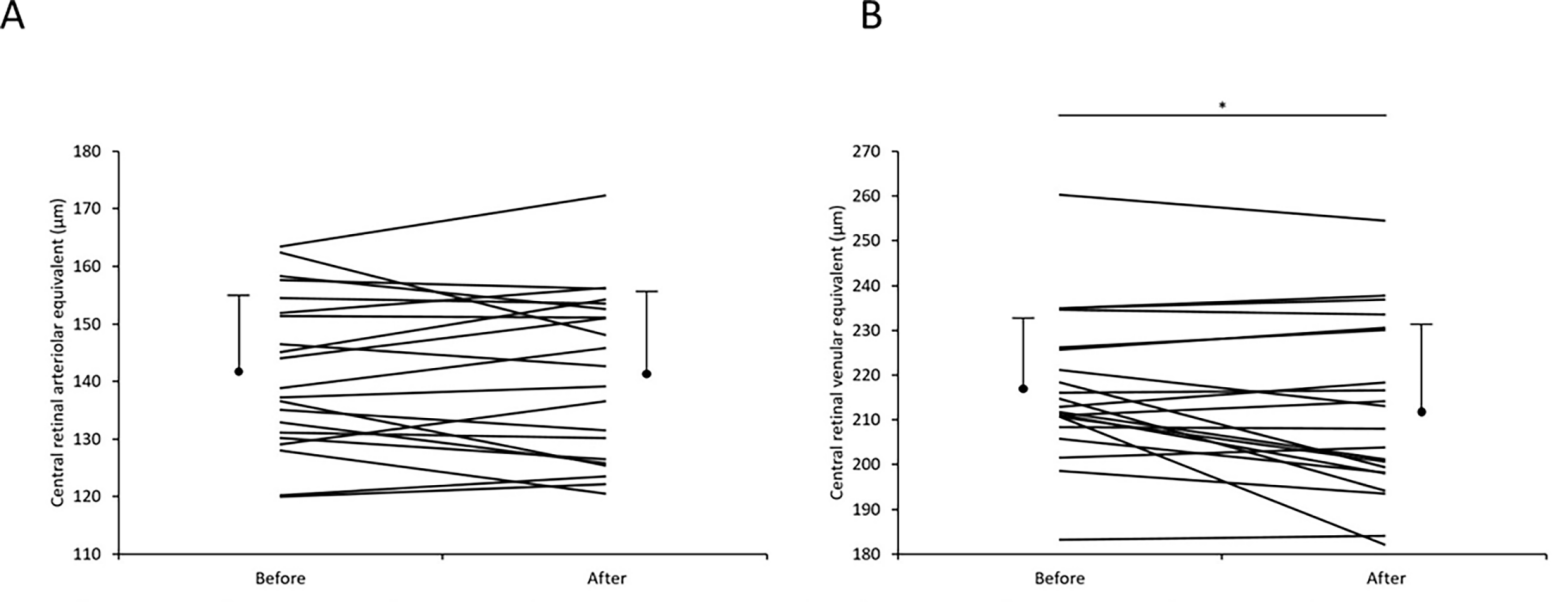
Calibre of arterioles and venules before and after coronary angiography in 21 subjects. **A.** Retinal arteriole calibre pre- and post-angiography; and **B.** Retinal venular calibre before and after coronary angiography. Means and SD are shown, * p = 0.02.

A cohort of 21 subjects were studied with retinal imaging both before and after angiography. Eighteen (86%) had a radial artery approach, 18 (86%) were administered intravenous verapamil, 19 (90%) had GTN, 19 (90%) had heparin, and their mean contrast load was 92.7 ±19.3 ml. Their average time to retinal imaging post- angiogram was 2.5 ± 0.7 hours. Retinal images were again deidentified, and calibre measurements performed at the retinal grading centre. These demonstrated that there was no difference in arteriolar calibre pre- and post-angiography, and a *reduction* in venular calibre (p = 0.02) **(Fig 1)**. No new hemorrhages were observed in the post-angiography images.

## Discussion

Population-based studies have found that venular dilatation is associated with increased cardiac events but a relationship of retinal microvascular abnormalities with coronary artery disease has been more difficult to demonstrate [21, 27]. However the present study suggests that microvascular abnormalities are associated with coronary artery disease: that retinal hemorrhage, and hence moderate microvascular retinopathy, correlates with a higher Leaman score and triple vessel disease; that retinal venular dilatation is associated with triple vessel disease; and that diabetic retinopathy is more common with worse Leaman and TIMI blush scores, and with LAD stenosis.

Most subjects undergoing angiography in this study had a microvascular retinopathy. This may have reflected their comorbid vascular risk factors such as age, gender, BP, diabetes, and smoking history [28]. Nearly half the subjects with diabetes had a background diabetic retinopathy but proliferative changes were less common.

Retinal hemorrhage occurred in just under one fifth of subjects. It occurs in both microvascular and diabetic retinopathy, and is not increased with aspirin or anticoagulant use [29]. No new hemorrhages were observed after angiography. Retinal hemorrhage was more common in subjects with more extensive and more severe coronary artery disease on Leaman score, possibly because of the shared associations with hypertension, diabetes and renal impairment. Retinal hemorrhage correlated with the number of diseased coronary vessels, but not with LAD stenosis in the Leaman score. The presence of microvascular retinopathy itself did not correlate with diseased vessel score nor worse LAD stenosis.

Arteriole calibre was not associated with any measure of coronary artery disease. However venular calibre was increased with triple vessel disease. This is consistent with the association of increased venular calibre with cardiac risk in epidemiological studies [14]. The vascular risk factors, diabetes, obesity, inflammation, dyslipidemia and smoking all contribute to venular dilatation [30], and may partly explain this association.

Moderate–proliferative diabetic retinopathy correlated with a higher Leaman score, independent of the traditional vascular risk factors. Diabetic retinopathy was also associated with an increased diseased vessel score, and with LAD stenosis ≥ 70% in both cases after adjusting for other vascular risk factors.

The TIMI blush score in part reflects small vessel disease, which is principally due to hypertension and diabetes. However microvascular retinopathy, as assessed here, was not associated with the TIMI blush score alone, but only after adjusting for vascular risk factors. Subjects with an abnormal TIMI blush score were more likely to be female, older, and have hypertension, diabetes, a smoking history, renal failure and anemia. Scoring of the TIMI blush score was a limitation of this study because a relatively subjective method was used, rather than the semiautomated methods now available.

Two previous studies compared retinal small vessel abnormalities and coronary artery disease [21, 27]. One examined 70 non-diabetic normotensive subjects and found that an abnormal ‘light reflex’, which usually reflects thickened atherosclerotic arterioles, was the most sensitive indicator of both the presence and severity of coronary artery disease. Narrowed and tortuous retinal vessels were less sensitive but more specific [27].

The results of the second report contrasted with ours. It concluded that retinal small vessel calibre was not associated with the Leaman score nor the number of diseased coronary vessels [21]. It did not examine the TIMI blush score nor other hypertensive microvascular features such as hemorrhage. This study comprised 98 subjects who resembled ours in age (median 64 years, 61 in ours), and prevalence of clinically- significant disease (62%, 70% in ours) but included fewer subjects with diabetes (27%, 38% in ours), and did not stipulate the numbers of women or smokers. These last factors are important because women, smokers, and subjects with diabetes, all have wider venular calibre [31–33], and excluding these groups meant a likely smaller mean venular calibre. This may explain why their study did not confirm the epidemiological observation that larger venules were associated with cardiac disease. The only methodological difference from our study was that retinal imaging was performed before angiography. This is unlikely to be important because we have demonstrated in supplementary experiments that arteriolar calibre was not different pre- and post-angiography despite the use of multiple vasoactive agents. This lack of effect may be explained by the short half-life of the medications [34–36].

A further study examined microvascular calibre in heart failure, not necessarily due to ischemic cardiac disease [37], and demonstrated increased arteriolar calibre. This result was more consistent with our findings since arteriolar and venular calibre are interdependent, and as one increases the other generally does too.

The strengths of our study were the large number of subjects, the high recruitment rate, the grading of coronary abnormalities by cardiologists, the methods of assessing microvascular and diabetic retinopathy, and the reproducibility of the calibre grading. The weaknesses were the single centre nature of the study, and the subjective nature of grading used for Leaman and TIMI blush scores.

Data on the association of retinal microvascular and diabetic abnormalities with coronary artery disease have been scarce. This study has demonstrated the utility of retinal hypertensive and diabetic microvascular examination in assessing coronary artery disease. Retinal imaging is an inexpensive non-invasive test that warrants further evaluation in identifying the presence, extent and nature of coronary artery disease.
